# Noninvasive Aortic Ultrafast Pulse Wave Velocity Associated With Framingham Risk Model: *in vivo* Feasibility Study

**DOI:** 10.3389/fcvm.2022.749098

**Published:** 2022-01-31

**Authors:** Jinbum Kang, Kanghee Han, Jihyun Hyung, Geu-Ru Hong, Yangmo Yoo

**Affiliations:** ^1^Deparment of Electronic Engineering, Sogang University, Seoul, South Korea; ^2^Division of Cardiology, Severance Cardiovascular Hospital, Yonsei University College of Medicine, Yonsei University Health System, Seoul, South Korea; ^3^Deparment of Biomedical Engineering, Sogang University, Seoul, South Korea

**Keywords:** arterial stiffness, framingham risk score (FRS), abdominal aorta, pulse wave velocity (PWV), ultrafast ultrasound imaging

## Abstract

**Background:**

Aortic pulse wave velocity (PWV) enables the direct assessment of aortic stiffness, which is an independent risk factor of cardiovascular (CV) events. The aim of this study is to evaluate the association between aortic PWV and CV risk model classified into three groups based on the Framingham risk score (FRS), i.e., low-risk (<10%), intermediate-risk (10~20%) and high-risk (>20%).

**Methods:**

To noninvasively estimate local PWV in an abdominal aorta, a high-spatiotemporal resolution PWV measurement method (>1 kHz) based on wide field-of-view ultrafast curved array imaging (ufcPWV) is proposed. In the ufcPWV measurement, a new aortic wall motion tracking algorithm based on adaptive reference frame update is performed to compensate errors from temporally accumulated out-of-plane motion. In addition, an aortic pressure waveform is simultaneously measured by applanation tonometry, and a theoretical PWV based on the Bramwell-Hill model (bhPWV) is derived. A total of 69 subjects (aged 23–86 years) according to the CV risk model were enrolled and examined with abdominal ultrasound scan.

**Results:**

The ufcPWV was significantly correlated with bhPWV (*r* = 0.847, *p* < 0.01), and it showed a statistically significant difference between low- and intermediate-risk groups (5.3 ± 1.1 vs. 8.3 ± 3.1 m/s, *p* < 0.01), and low- and high-risk groups (5.3 ± 1.1 vs. 10.8 ± 2.5 m/s, *p* < 0.01) while there is no significant difference between intermediate- and high-risk groups (8.3 ± 3.1 vs. 10.8 ± 2.5 m/s, *p* = 0.121). Moreover, it showed a significant difference between two evaluation groups [low- (<10%) vs. higher-risk group (≥10%)] (5.3 ± 1.1 vs. 9.4 ± 3.1 m/s, *p* < 0.01) when the intermediate- and high-risk groups were merged into a higher-risk group.

**Conclusion:**

This feasibility study based on CV risk model demonstrated that the aortic ufcPWV measurement has the potential to be a new approach to overcome the limitations of conventional systemic measurement methods in the assessment of aortic stiffness.

## Introduction

The elasticity of proximal large arteries is determined by a high elastin to collagen ratio, and the increase in arterial stiffness is mostly caused by the progressive elastic fiber degeneration (1, 2). The physical stiffening of arteries eventually increases the risk of cardiovascular (CV) disease, such as systolic hypertension, coronary artery disease, myocardial infarction and stroke (3–5). Aortic pulse wave velocity (PWV) has been considered as one of the most reliable clinical parameters for evaluating arterial stiffness, blood pressure, therapeutic efficacy and CV risk stratification in patients (6, 7). It depends not only on structural changes associated with the elastic modulus of the wall affecting wave propagation but also on aortic pressure, which has a strong direct relationship to stiffness (3).

The PWV is defined by the speed at which a forward pressure wave is transmitted from the aorta through the vascular tree. To estimate PWV (e.g., a few meters per second), the propagating distance and the time of the arterial waveform passing between the two sites are measured, and the carotid-femoral PWV is currently being regarded as the gold standard method (8–10). However, these systemic or regional PWV measurements cannot accurately assess biomechanical properties of vessel segments so that the invasive methods using a pressure catheter are still required for the assessment of local aortic compliance (11, 12). Therefore, several local based PWV measurement techniques have been introduced to noninvasively evaluate aortic segments along the arterial tree.

Cardiovascular magnetic resonance (CMR) based PWV assessment substantially reduces errors by using accurate aortic length and transit times between flow waves (13, 14). Doppler ultrasound or pulse wave imaging with electrocardiogram (ECG) synchronization allows the estimation of PWV values using the time delay between two close positions during a cardiac cycle along the vessels (15–17). However, these approaches suffer from a relatively low frame rate compared to PWV. To improve accuracy diminished by limited temporal resolution, a plane wave transmission based ultrafast PWV measurement method was recently proposed and it showed a real-time direct measurement of local PWV with high spatiotemporal resolution (18–21). With the advantages of simplicity and accessibility, it has been extensively studied for various clinical usages, such as carotid stiffness assessment (22–27).

From the previous studies, a local PWV measurement technique based on high spatiotemporal resolution is strongly required, and the ultrafast PWV measurement for aortic segments in human abdomen are still rarely studied due to low accessibility and technical limitations, such as a deep and wide field-of-view (FOV). Here, we propose a high spatiotemporal resolution aortic PWV measurement method based on wide FOV ultrafast curved array imaging (28) using diverging wave transmission (ufcPWV). In addition, a new aortic wall motion tracking algorithm based on adaptive reference frame update was also conducted to compensate errors from temporally accumulated out-of-plane motion. Using the proposed method, a feasibility study to investigate the correlation between PWV for abdominal aorta and a CV risk model was performed. The aortic central pressure waveform was simultaneously measured by the applanation tonometer, and a theoretical PWV based on Bramwell-Hill model (bhPWV) (29) was derived. The CV risk model was classified into three groups based on the Framingham risk score (FRS), which is one of the scoring systems used to estimate the 10-year CV risk (30), i.e., low-risk (<10%), intermediate-risk (10~20%) and high-risk (>20%). We hypothesized that aortic ufcPWV is associated with the Framingham risk model.

## Materials and Methods

### Study Protocol

The CV risk model was classified into three groups based on the Framingham risk score (FRS), i.e., low-risk (<10%), intermediate-risk (10~20%) and high-risk (>20%). The study was approved by the Institutional Review Board of the Clinical Trials Center of Yonsei University Health System, and the written informed consent was obtained from all patients. 31 patients for each risk group (total 93 patients) were recruited, but a few patients in the intermediate- and high-risk groups were excluded due to the poor image quality or incomplete scanning of the abdominal aorta. The study population's characteristics of the remaining 69 patients were presented in Table 1. The population contained a wide range of age, i.e., 23–86 years, and the heart rate and blood pressure were within normal range. FRS showed a significant difference between each risk groups. Abdominal ultrasound scans in longitudinal and transverse views were performed by the commercialized ultrasound research platform (E-Cube 12R, Alpinion Medical Systems Co., Ltd., Anyang-si, Gyeonggi-do, Korea) using a convex array transducer (C1-6, Alpinion Medical Systems Co., Ltd., Anyang-si, Gyeonggi-do, Korea). The radiofrequency (RF) data of three cardiac cycles were captured based on a real-time ultrafast curved array imaging (28) at a frame rate of 1 kHz. The acoustical energy was measured and set under FDA safety limit (mechanical index (MI) <1.9 and spatial peak time average intensity (Ispta) <720 mW/cm^2^).

**Table 1:** Baseline characteristics and measurements of the study population (*n* = 69) classified by Framingham risk model.

	**All (*n* = 69)**	**Low-risk (*n* = 31)**	**Intermediate-risk (*n* = 22)**	**High-risk (*n* = 16)**	***p***
Sex, male/female	48/21	18/13	14/8	16/0	
Age, years	54.0 **±** 18.1	40.5 **±** 16.2	61.9 **±** 10.9^a^	69.1 **±** 8.9^b^	<0.001
Body mass index, kg/m^2^	24.0 **±** 2.8	23.2 **±** 3.0	24.8 **±** 2.8	24.6 **±** 2.0	0.105
Heart rate, beats/min	75.0 **±** 11.7	74.1 **±** 12.3	75.5 **±** 10.1	73.9 **±** 13.1	0.891
Systolic blood pressure, mmHg	126.6 **±** 15.4	124.7 **±** 17.2	128.8 **±** 10.7	127.0 **±** 17.6	0.635
Diastolic blood pressure, mmHg	76.3 **±** 11.1	74.9 **±** 11.7	78.5 **±** 10.5	76.1 **±** 11.0	0.519
Total cholesterol, mg/dL	169.0 **±** 38.9	169.7 **±** 31.1	167.9 **±** 49.3	168.8 **±** 39.1	0.986
HDL cholesterol, mg/dL	53.5 **±** 13.0	54.5 **±** 12.5	55.0 **±** 13.2	49.7 **±** 13.6	0.407
Hypertension, *n* (%)	39 (57%)	7 (23%)	20 (91%) ^a^	12 (75%) ^b^	<0.001
Current smoker, *n* (%)	10 (14%)	4 (13%)	3 (14%)	3 (19%)	0.862
Diabetes mellitus, *n* (%)	16 (23%)	0 (0%)	4 (18%)	12 (75%)^b,c^	<0.001
FRS	14.4 **±** 13.9	3.6 **±** 2.5	13.9 **±** 2.6^a^	36.1 **±** 10.5^b,c^	<0.001

*FRS, Framingham risk score*.

*The subjects were classified into three risk groups according to FRSs: low risk (<10%), intermediate risk (10–20%), and high risk (>20%)*.

*Values are means ± SD*.

a *Statistically significant difference (p < 0.05) between Low risk and Intermediate risk after Bonferroni correction*.

b *Statistically significant difference (p < 0.05) between Low risk and High risk after Bonferroni correction*.

c *Statistically significant difference (p < 0.05) between Intermediate risk and High risk after Bonferroni correction*.

### A Real-Time Ultrafast Curved Array Imaging

**Figure 1:**
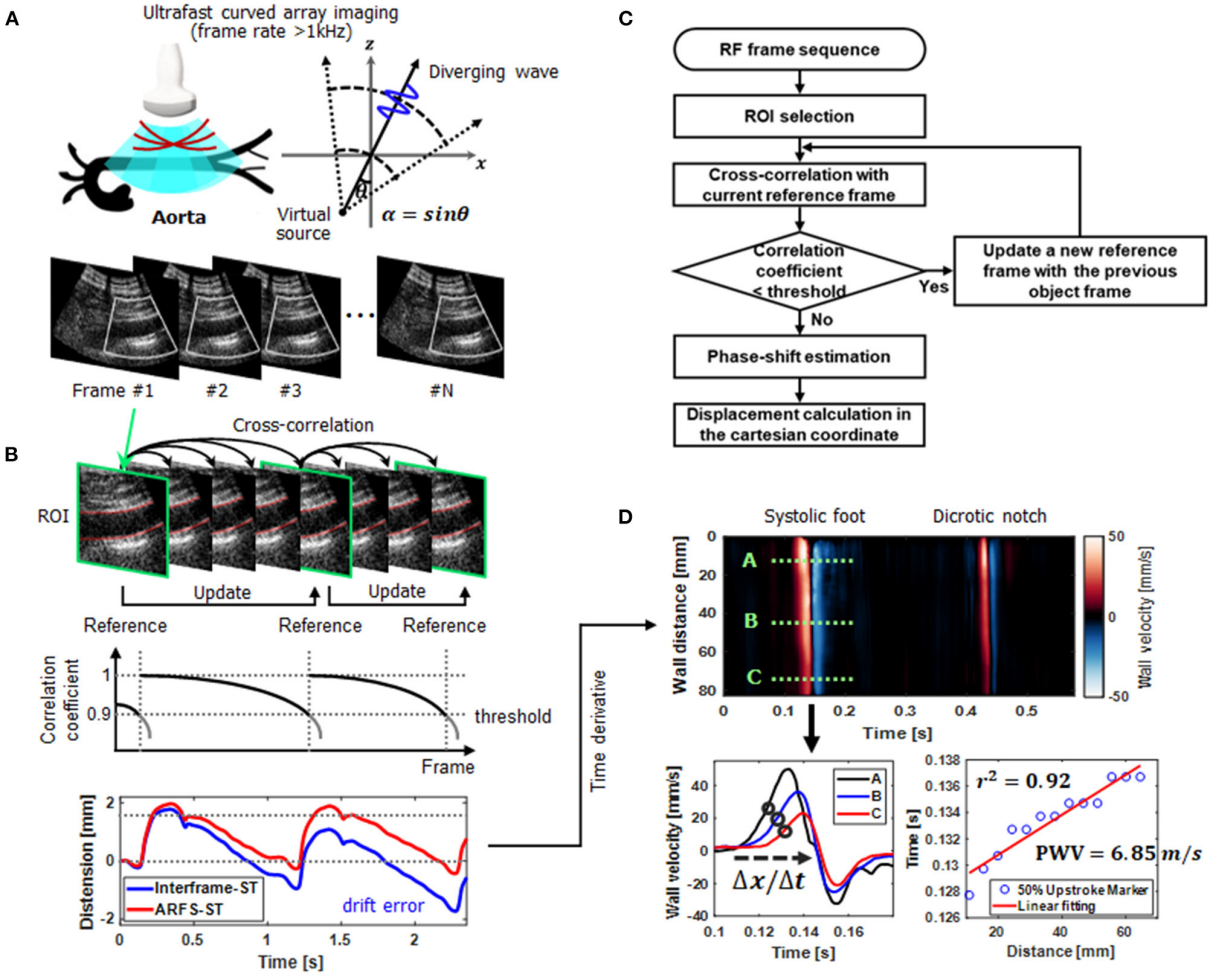
An overall schematic representation of aortic ultrafast pulse wave velocity (PWV) assessment procedure. **(A)** Real-time scanning and a full scanline RF data acquisition using ultrafast curved array imaging based on diverging wave transmissions. For a diverging wave transmission, the virtual source was located in a circular line with the radius of the curved array transducer and the steering angle (θ). **(B)** Aortic wall motion tracking (speckle correlation) based on a new adaptive reference frame update (ARFD) method. By dynamically updating the reference frame in accordance with the correlation coefficient, the intrinsic error or bias (e.g., drift error) from out-of-plane motion or scanning is compensated. **(C)** The flow chart of the ARFD implementation. **(D)** Aortic ultrafast PWV (ufcPWV) imaging and calculation (PWV = Δdistance/Δtime) based on the time-distance plot for the 50% upstroke marker of the forward wave.

To assess pulse wave velocities (PWVs) in aortas, the recently proposed ultrafast curved array imaging technique (28), which provides high spatiotemporal resolution with a wide field-of-view (FOV) for abdominal applications, was employed as illustrated in Figure 1A. For a diverging wave transmission, the virtual source was located in a circular line with the radius of the curved array transducer to obtain a wide FOV and 3-tilted diverging waves in linear increments ranging from −12 to +12 were utilized (the frame rate >1 kHz). The ultrafast curved array imaging based on diverging wave transmissions was implemented in the ultrasound research scanner for a real-time scanning and a full scanline RF data acquisition.

### Adaptive Reference Frame Update Based Speckle Correlation

For PWV measurements, vessel wall motion tracking (speckle correlation) is usually conducted, and the selection of a reference frame is crucial to accurately estimate arterial wall motion. In addition, the out-of-phase motion or unexpected noise may disrupt highly accurate motion estimation, and the unwanted errors (e.g., drift errors) are typically accumulated with a common fixed reference or interframe-based motion estimation methods (31–33). As illustrated in Figure 1B, to improve the performance of vessel wall motion tracking, an adaptive reference frame update (ARFD)-based speckle correlation is proposed, and the flow chart of the proposed ARFD is described in Figure 1C. In the ARFD, the reference frame is dynamically updated in accordance with the correlation coefficient determined by a threshold value (e.g., 0.9 in Figure 1B). By applying the proposed ARFD method to the vessel wall motion estimation, the intrinsic error or bias from out-of-plane motion or scanning can be properly compensated, and it enables more robust Lagrangian speckle correlation (34) with a minimal accumulation of errors, as shown in Figure 1B. For vessel wall motion tracking, the axial displacement in the anterior or posterior wall was measured by using the time domain phase shift estimation based on the 1-D cross-correlation. The window size of 8λ was used for the motion tracking within the search range of 9 times the window size, and the window overlap was 90%.

### Pulse Wave Velocity Imaging and Measurement

Based on the ultrafast curved array imaging and ARFD-based speckle correlation algorithm, the aortic ultrafast PWV (ufcPWV) imaging and measurement was conducted. To do this, a high-spatiotemporal resolution pulse wave-induced wall displacement was estimated. Anterior or posterior vessel wall segmentation was first performed, and the distance of the vessel wall was calculated. Then, the wall velocity waveform was derived from the estimated displacement using a 9-point Savitzky-Golay digital differentiator for the temporal derivative (35). Therefore, the 2-D spatiotemporal wall velocity variation map (i.e., PWV imaging), which depicts the pulse wave propagation, was produced for a PWV measurement, as illustrated in Figure 1D. To measure a PWV along the vessels, the time-distance plot was first generated using the 50% upstroke points of the forward wave (e.g., the black circles on A, B and C velocity waveforms in Figure 1D) (systolic foot) as the tracking feature in the wall velocity waveform. Then, a linear regression fitting on the detected upstroke points was conducted for PWV calculation (PWV = Δdistance/Δtime) as described in Figure 1D. The PWVs of three cardiac cycles (i.e., data acquisition time of 3.0 sec) were measured and averaged for each case.

### Local Pulse Wave Velocity Measurement by Bramwell-Hill Equation

To investigate the association and agreement between the direct PWV measurement (ufcPWV) and theoretical PWVs derived from arterial distensibility, a local aortic PWV was obtained with the Bramwell-Hill equation (bhPWV) (36). The Bramwell-Hill model describes the relation between vascular wall stiffness expressed in arterial distensibility and the PWV. Aortic distensibility can be defined as the relative change in vessel diameter over local pulse pressure (29):


(1) $$\begin{array}{r} \text{bhPWV}=\sqrt{\frac{{\text{A}}_{\text{d}}\cdot \text{PP}}{\rho \cdot \Delta \text{A}}} \end{array}$$

where *A*_*d*_ is the cross-sectional area in diastole, and *A* is the difference of the cross-sectional area between systole and diastole in the cardiac cycle. To measure the cross-sectional area of aortic vessel, the envelope image in the transversal view was utilized and the diameter was calculated assuming a circular shape. ρ is the blood density (1060 kg/m^3^), and *PP* is the local pulse pressure. To noninvasively measure the local *PP* in aorta, the arterial applanation tonometry (SphygmoCor, AtCor Medical, Sydney, NSW, Australia) was performed and the central blood pressure waveform was obtained.

### Data Analysis

Data from a total of 69 patients were processed, and a statistical analysis was performed to examine differences between each risk groups. The initial three evaluation groups were further classified into two evaluation groups by merging the intermediate- and the high-risk groups into a higher-risk group [i.e., low-risk (<10%, *n* = 31) and higher-risk (≥10%, *n* = 38)]. The signal and image processing were externally conducted with MATLAB R2019b (The MathWorks, Natick, Massachusetts, USA). The coefficient of determination (*r*^2^) in linear regression was evaluated to indicate the reliability of PWV calculation (Figure 1D) (37). The statistical data assessment was also conducted with the statistics analysis software (SPSS, IBM, Armonk, NY, USA). For the three evaluation groups, the Kruskal-Wallis one-way analysis of variance (ANOVA) test was performed in accordance with normality and homogeneity of variance, and a significance probability was corrected by the Bonferroni *post-hoc* analysis. To demonstrate a difference between two evaluation groups, the Welch's *t*-test was conducted in the similar way with the three evaluation groups. The association and agreement between ufcPWV and bhPWV were also analyzed and the relationships were determined by calculating the Pearson's correlation coefficient (*r*).

## Results

### Correlation Between ufcPWV and bhPWV Measurement

**Figure 2:**
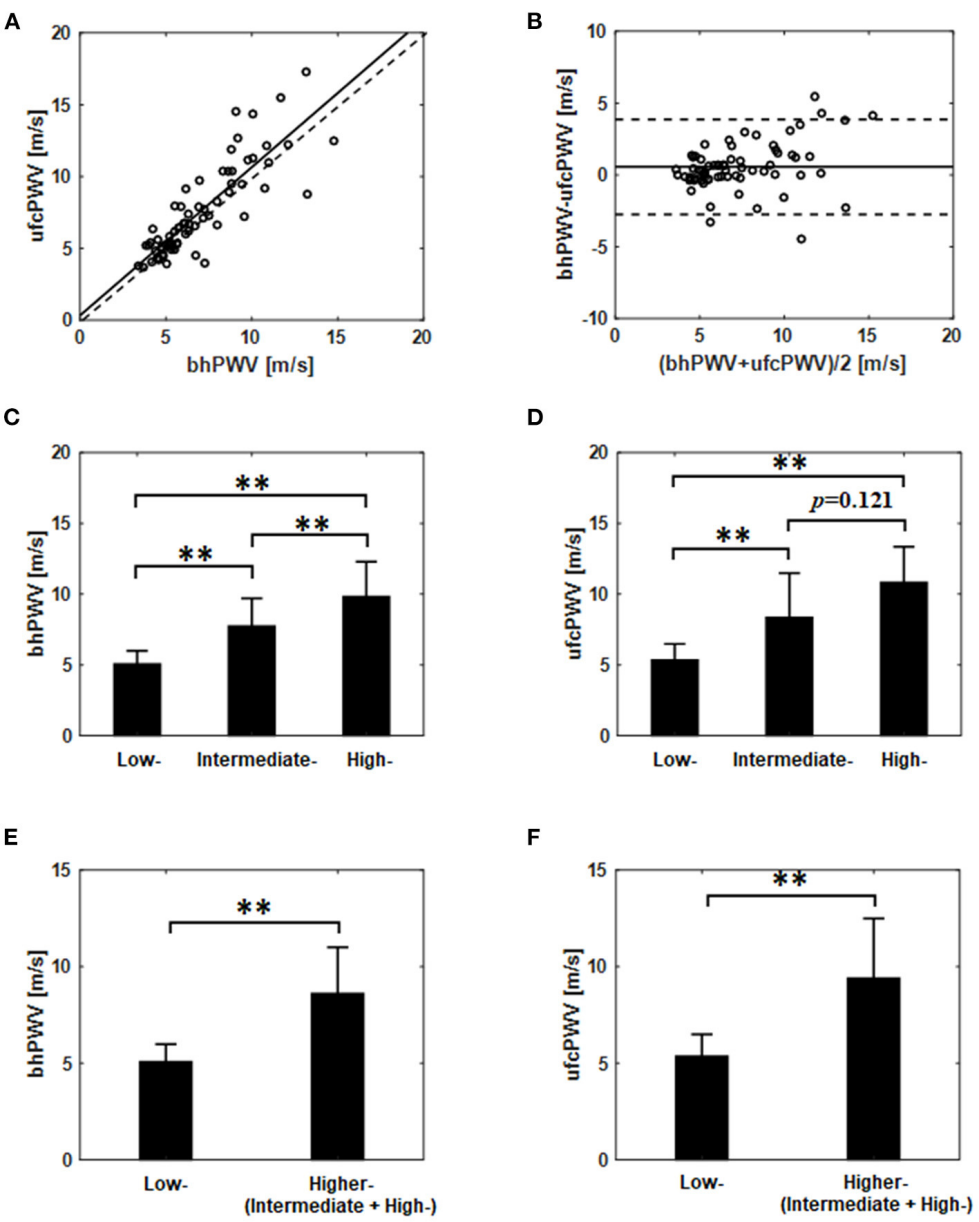
**(A)** Correlation and **(B)** agreement assessment between the ufcPWV based on the ultrafast curved array imaging and the bhPWV measured via Bramwell-Hill equation (solid line indicates the mean difference between the two measurements and dashed lines represent mean ± 1.96SD). Comparisons between the three evaluation groups [i.e., low- (<10%), intermediate- (10~20%) and high-risk (>20%)] based on Framingham risk score from **(C)** the bhPWV and **(D)** the ufcPWV measurement. Comparisons between the two evaluation groups [i.e., low- (<10%) vs. higher-risk (≥10%)] from **(E)** the bhPWV and **(F)** the ufcPWV measurement. ** indicates *p* < 0.01.

The association between the ufcPWV and the bhPWV was evaluated for all subjects (*n* = 69). As illustrated in Figure 2A, the ufcPWV was significantly correlated with bhPWV measured via Bramwell-Hill model (*r* = 0.85, *p* < 0.01). Figure 2B represents the Bland-Altman plot to assess the agreement between the ufcPWV and the bhPWV, and they showed a nonsignificant difference between the two measurements (the limits of agreements: −2.7–3.8 m/s).

### Comparison Between CV Risk Groups

In the analysis of the three evaluation groups (low- (*n* = 31), intermediate- (*n* = 22) and high-risk (*n* = 16); Table 1) according to Framingham risk score, the bhPWV showed a statistically significant difference, i.e., low- and intermediate-risk (5.1 ± 0.9 vs. 7.7 ± 2.0 m/s, *p* < 0.01), low- and high-risk groups (5.1 ± 0.9 vs. 9.8 ± 2.5 m/s, *p* < 0.01) and intermediate- and high-risk group (7.7 ± 2.0 vs. 9.8 ± 2.5 m/s, *p* < 0.01), respectively, as illustrated in Figure 2C. For the ufcPWV measurement, as illustrated in Figure 2D, it showed a statistically significant difference between low- and intermediate-risk (5.3 ± 1.1 vs. 8.3 ± 3.1 m/s, *p* < 0.01), and low- and high-risk groups (5.3 ± 1.1 vs. 10.8 ± 2.5 m/s, *p* < 0.01) while there is no significant difference between intermediate- and high-risk group (8.3±3.1 vs. 10.8±2.5 m/s, *p*=0.121).

To further analyze the differences between the CV risk models, two modified evaluation groups consisting of the low-risk group (<10%, *n* = 31) and the higher-risk group (≥10%, *n* = 38; the intermediate and the high-risk), were analyzed in the same manner. Figures 2E,F show the comparison results using the ufcPWV based on the ultrafast curved array imaging and the bhPWV measured via the Bramwell-Hill equation, respectively. As illustrated in Figures 2E,F, there was a statistically significant difference between low- and higher-risk group in both the bhPWV (5.1 ± 0.9 vs. 8.6 ± 2.4 m/s, *p* < 0.01) and the ufcPWV measurements (5.3 ± 1.1 vs. 9.4 ± 3.1 m/s, *p* < 0.01).

### Association of ufcPWV With Framingham Risk Score

**Figure 3:**
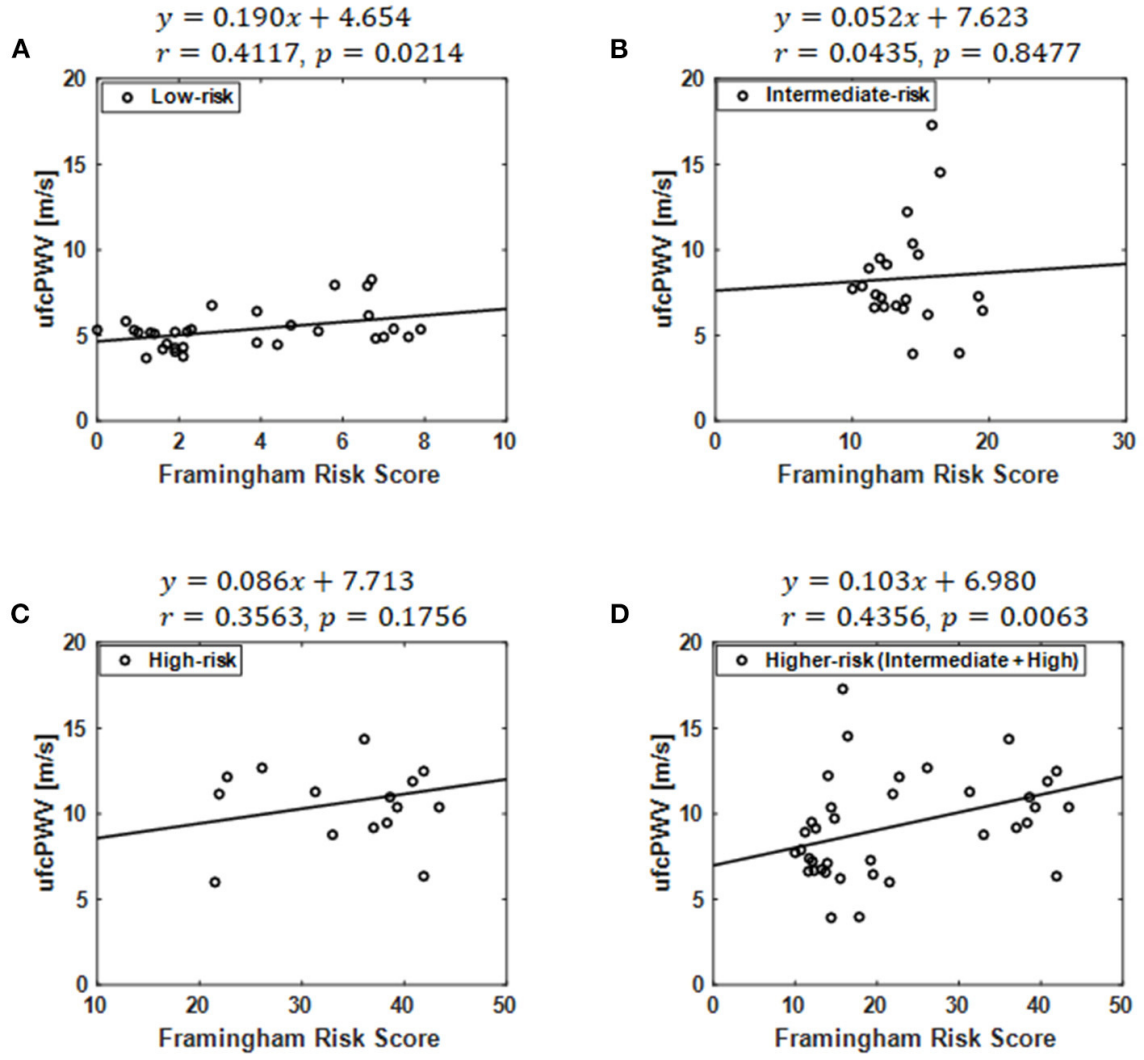
Correlation between the ufcPWV based on ultrafast curved array imaging and Framingham risk score in the case of **(A)** the low-risk model, **(B)** the intermediate-risk model, **(C)** the high-risk model and **(D)** the higher-risk model combined with the intermediate and the high-risk groups.

A significant correlation was found between the ufcPWV and FRS (*r* = 0.41, *p* < 0.05; Figure 3A) in the low-risk case while there were no correlation in the intermediate- and high-risk groups, as shown in Figures 3B,C. The linear fitting between the ufcPWV and FRS in the low-risk case showed an increase of 0.19 m/s in ufcPWV per 1-FRS. Moreover, the ufcPWV in the case of the higher-risk group combined with the intermediate and the high-risk subjects was also significantly correlated with the FRS (*r* = 0.44, *p* < 0.01) as described in Figure 3D, and the linear fitting indicated an increase of 1.0 m/s per 10-FRS.

### Association of ufcPWV With Systolic Blood Pressure

**Figure 4:**
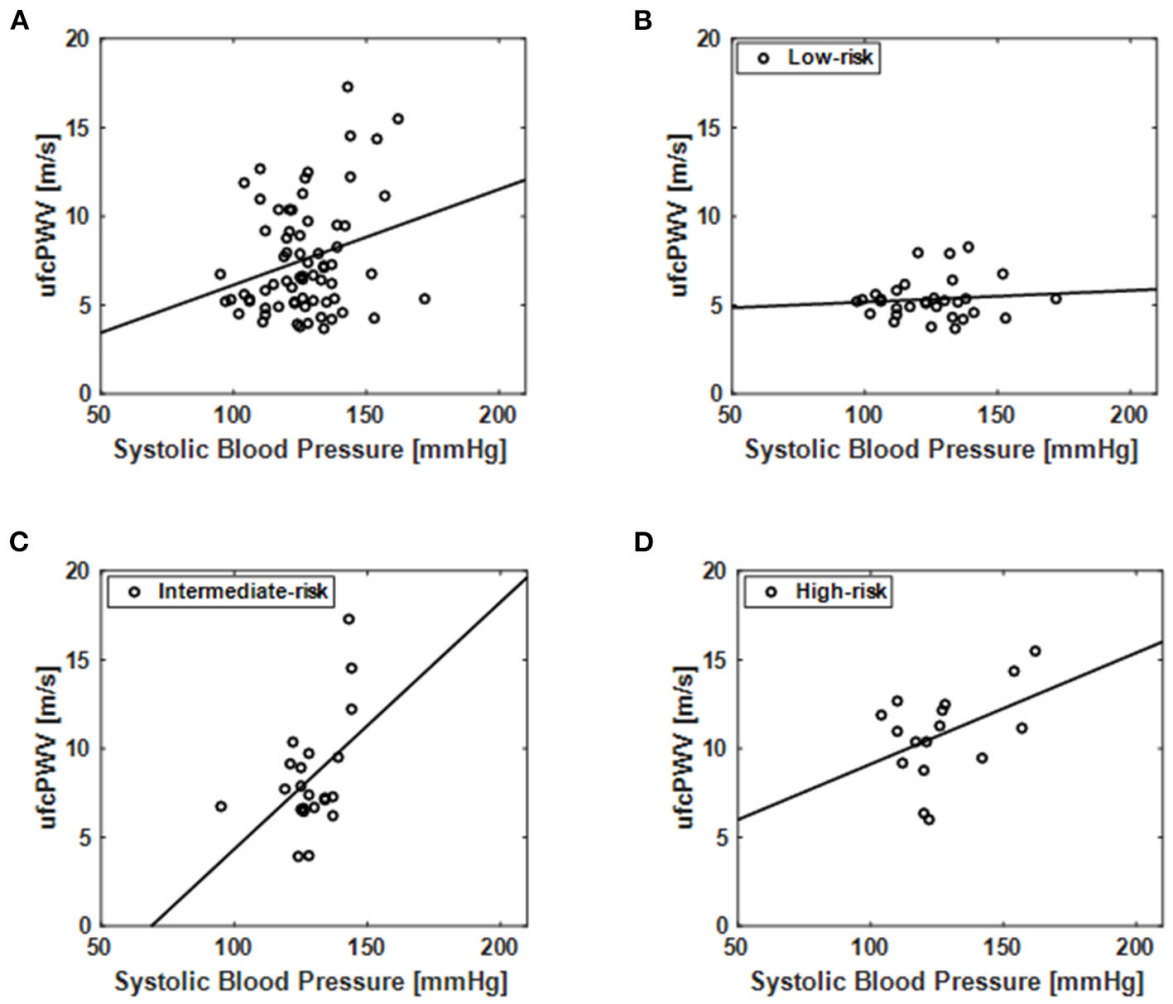
Correlation of the ufcPWV based on ultrafast curved array imaging with systolic blood pressure (Table 1) for **(A)** all subjects (*n* = 69) and individual CV risk groups [**(B)** low-, **(C)** intermediate- and **(D)** high-risk].

The relation of ufcPWV to systolic blood pressure (Table 1), which is a classic measure of arterial stiffness, was additionally assessed, as illustrated in Figure 4. Figure 4A represents the relation between ufcPWV and systolic blood pressure for all subjects (*n* = 69), and it showed a significant correlation (*r* = 0.26, *p* < 0.05) between the two clinical parameters. To further analyze the association according to the CV risk model, systolic blood pressures for the three evaluation groups were respectively evaluated. As shown in Figures 4B–D, the ufcPWV was significantly correlated with the systolic blood pressure in the intermediate and high-risk groups, respectively, (*r* = 0.47 and *r* = 0.45, all *p* < 0.05) while the low-risk group showed no significant association (*r* = 0.10, *p* = 0.60).

## Discussion

Pulse waves are propagated through arteries, and PWV is affected by the mechanical properties in the pathological process of arterial wall changes along the arterial system. Therefore, PWV is not constant and varies from one location to another since the geometrical and mechanical properties vary along the arterial tree, and there are differences between central elastic arteries and more peripheral, muscular arteries. For these reasons, the importance of measuring local PWV is increasing in contrast to measuring systemic PWV, which can only be estimated an averaged PWV. For example, aortic PWV, which is one of the most reliable clinical parameters for evaluating arterial stiffness, has been generally measured in two different sites (e.g., carotid and femoral arteries). The carotid-femoral PWV measurement is currently accepted as the gold-standard technique for arterial stiffness assessments. For carotid-femoral PWV measurements, several tonometry techniques based on pressure sensors with ECG gating (e.g., PulsePen, Complior and SphygmoCor) have been widely used in research and clinical settings (11). However, distance measurements of the pulse wave pathway are approximately estimated with the sensor location on the body surface, thus causing a crucial systemic error in the PWV measurements. To overcome this limitation, local PWV measurement methods through direct visualization of local vessels have been introduced based on ultrasound or MRI imaging techniques. The ultrasound technique allows the determination of the PWV by estimating the time delay between the diameter waveforms at two close positions in a local site, e.g., Doppler-derived PWV, flow-area method and pulse wave imaging (15–17, 38). The MRI-based PWV estimation uses the accurate and direct measurement of the path length of pulse waves between two imaging levels and provides aortic vascular parameters (aortic distensibility, aortic compliance, aortic elastic modulus and aortic stiffness index) (39, 40). These imaging-guided PWV measurements take advantage of the direct measurement for the pulse wave pathway and can avoid the systemic error induced by the coarse estimation of distance.

A major advantage of the local measurement of PWV is that a direct measurement of local vessels is strongly related to wall stiffness. In the early stage of arterial stiffness, the elastic properties are affected by locally scattered fibrous spots on the arterial wall, and the heterogeneous structure of arterial walls at different sites produces different functional properties between vessel segments. Regarding strongly localized wall heterogeneity, arterial wall mechanical properties can be dramatically altered within a small region (from a few millimeters to a few centimeters), e.g., abdominal aortic aneurysms or arterial plaques. However, the most current PWV measurements (e.g., carotid-femoral PWV) cannot assess biomechanical properties in local vessels, and they cannot directly evaluate the arterial stiffness of vessel segments due to the accessibility and the limited temporal resolution of the techniques. The temporal resolution (i.e., frame-rate) is the most significant technical factor in the PWV measurements. To properly snapshot the accelerated pulse wave (5~10 m/s), data acquisition rates must be increased relative to the PWV. However, the accuracy and reliability of the PWV measurement approaches usually suffer from an inherent technical tradeoff between spatial resolution (image quality) and temporal resolution (frame rate). Inaccurate measurements imposed by frame-rate limitations can increase errors as the PWV increases by arterial stiffening. Therefore, a high-frame-rate-based accurate PWV measurement technique is still required.

The FRS, which estimates the 10-year CV risk of an individual, is calculated based on a variety of risk factors such as age, smoking history, diabetes mellitus, systolic blood pressure, HDL-C concentration. The FRS is considered as a useful tool for quantitative assessment of the risk for CV disease in the general populations (30, 41–43) and it can be closely related to PWV since both FRS and PWV are widely used as surrogate markers to predict future CV disease by quantifying total CV risk. However, the correlation between FRS and PWV is rarely reported although it has potentials to develop into a more powerful biomarker for CV risk prediction (6, 44–46).

In this article, we investigated the association between the Framingham risk model classified with FRS and aortic PWV. To measure local PWV in a specific aortic segment (i.e., abdominal aorta) with high spatiotemporal resolution (>1 kHz), wide field-of-view ultrafast curved array imaging based PWV measurement method (ufcPWV) was proposed (Figure 1). In the result for the three risk groups (Figure 2), the ufcPWV showed a statistically significant difference between low- and intermediate-risk, and low- and high-risk groups, but there is no significant difference between intermediate- and high-risk groups. To compensate this factor, two evaluation groups consisting of the low- (FRS <10%) and the higher-risk group (FRS ≥ 10%) were additionally assessed, and it showed a highly significant difference in both bhPWV and ufcPWV (Figure 2). The additional analysis to investigate the direct correlation between FRS and ufcPWV was performed, and only the low- and the higher-risk groups showed a significant correlation with different increments of PWV (Figure 3). The direct association between FRS and aortic PWV should be more investigated with large populations.

The association between aortic PWV and systolic blood pressure among the clinical parameters (Table 1) was additionally evaluated since the systolic blood pressure is one of the mostly contributing factors to PWV (47). In the assessment for all subjects, the ufcPWV and systolic blood pressure showed a significant relationship, as illustrated in Figure 4A. However, in the analysis according to the CV risk model, only the intermediate and high-risk groups showed a significant association between the two parameters although the groups of the systolic blood pressure showed no significant differences (Table 1). It means that aortic PWV in higher risk group may be correlated with CV risk independently of the systolic blood pressure, but it should be investigated with more obvious control groups as well as large populations.

One of the limitations of this study is the lack of comparison with carotid-femoral PWV based on tonometry, which is the most validated technique to estimate arterial stiffness and correlated with CV risk (48). A validation study between carotid-femoral PWV and local ufcPWV may further improve an aortic PWV as a useful biomarker for predicting CV risk. The other limitation is that any tags for the same scanning site (i.e., abdominal aorta) were not utilized during examinations although patients in the supine position were basically scanned by experienced sonographers. In addition, there was no assessment of intra- and inter-observer variability in PWV measurements. A number of data sets in the intermediate- (*n* = 9) and the high-risk (*n* = 15) groups (Table 1) were excluded due to poor image quality or a history of CV disease. The main cause for the deterioration of image quality may be due to the degradation in receive beamforming since a thick layer of fat in obese patients incurs severe phase aberration. Furthermore, linear regression with *r*^2^ < 0.5 was considered unreliable and the corresponding PWV estimate would be rejected.

For the future PWV measurements, relatively inexpensive techniques with fewer approximations, leading to an accurate evaluation, will be needed for the more efficient diagnostic tool in detecting CV diseases in early stages. In addition, highly accurate PWV measurements and direct arterial stiffness assessments may also improve the management of the process of CV risks or the monitoring of therapy in patients with conditions such as isolated systolic hypertension. In future work, aortic PWVs in different segmental regions (e.g., ascending aorta, arch of aorta and descending aorta) will be measured and evaluated in various clinical settings (e.g., atherosclerosis).

## Conclusion

In this paper, a high-spatiotemporal resolution aortic PWV measurement method based on ultrafast curved array imaging (ufcPWV) was proposed and it showed the association with Framingham risk model. This feasibility study demonstrated that the ufcPWV measurement has the potential to be a new approach to overcome the limitations of conventional systemic measurement methods in the assessment of aortic stiffness.

## Data Availability Statement

The original contributions presented in the study are included in the article/supplementary material, further inquiries can be directed to the corresponding author.

## Ethics Statement

The studies involving human participants were reviewed and approved by Institutional Review Board of the Clinical Trials Center of Yonsei University Health System (IRB number: 1-2019-0065). The patients/participants provided their written informed consent to participate in this study.

## Author Contributions

JK and KH performed the theoretical and experimental analyses and implementation of *the in vivo* study. JH and G-RH designed the clinical study protocol and obtained data from patients. G-RH and YY supervised the study. JK, G-RH, and YY wrote the manuscript. All authors contributed to the article and approved the submitted version.

## Funding

This work was supported by the National Research Foundation of Korea (NRF) grant funded by the Korean government (MSIT) (NRF-2021R1A2C3006264) and the Korea Medical Device Development Fund grant funded by the Korea government (the Ministry of Science and ICT, the Ministry of Trade, Industry and Energy, the Ministry of Health & Welfare, and the Ministry of Food and Drug Safety) (Project Number: 202011A01).

## Conflict of Interest

The authors declare that the research was conducted in the absence of any commercial or financial relationships that could be construed as a potential conflict of interest.

## Publisher's Note

All claims expressed in this article are solely those of the authors and do not necessarily represent those of their affiliated organizations, or those of the publisher, the editors and the reviewers. Any product that may be evaluated in this article, or claim that may be made by its manufacturer, is not guaranteed or endorsed by the publisher.

## Abbreviations

PWV: pulse wave velocity
CV: cardiovascular
FRS: Framingham risk score
ECG: electrocardiogram
FOV: field-of-view
CMR: Cardiovascular magnetic resonance
ufcPWV: ultrafast curved array imaging
bhPWV: theoretical PWV based on the Bramwell-Hill model.
